# Editorial: Innovative Imaging Techniques in Preclinical Models of Neurodegenerative Diseases

**DOI:** 10.3389/fnins.2021.801037

**Published:** 2021-12-23

**Authors:** Rodolfo G. Gatto, Yu-Chien Wu

**Affiliations:** ^1^Department of Bioengineering, University of Illinois at Chicago, Chicago, IL, United States; ^2^Department of Radiology and Imaging Sciences, Indiana University School of Medicine, Indianapolis, IN, United States

**Keywords:** neuroimaging technologies, neurodegenerative diseases, preclinical animal models, neuroscience methods, axonal connectivity

Neurodegenerative disorders have been forecast as the next global pandemic. Besides the growing understanding of the basic molecular mechanisms associated to neurodegenerative diseases (NDDs), the current number of disease-modifying treatments remains quite limited. Considering the high social impact among such diseases, technical resources have been focused on the investigation of early biomarkers aiming to maximize the treatment exposures and improve the patient's prognosis.

Improvements in imaging systems and more precise genetic manipulations in biological models are possible new routes toward quantifying diseases progression or developing disease-modifying therapy. In invertebrate models, such as the fruit fly, the compound eye is a premier experimental system for modeling human neurodegenerative diseases. The disruption of the retinal geometry has been historically assessed using time-consuming and poorly reliable techniques such as histology manual counting. Recent semiautomated quantification approaches rely either on manual region-of-interest delimitation or automated methods to estimate the extent of degeneration. The work from Diez-Hermano et al. presents a fully automated classification pipeline of bright-field images, based on orientated gradient descriptors and machine learning (ML) techniques. As an example, the author's initial region-of-interest (ROI) extraction was performed applying image classification algorithms by different ML approaches on independent datasets (Diez-Hermano et al.). Therefore, the authors proved ML as a useful tool to combine imaging techniques in the early detection of AD and screen for mild cognitive impairment (MCI). On the other side, the work of Pan et al. used convolutional neural network (CNN) taking advantage to its excellent efficiency in automated feature learning from a variety of multilayer perceptrons. As such, ensemble learning (EL) has shown robustness as a learning-system performance via multi-model integration. Therefore, combining CNN and EL on a set of MRI images, the authors were able to identify subjects with MCI or AD (Pan et al.).

Understanding non-human microstructural brain alterations in the course of neurodegenerative diseases (NDDs) has substantially improved by incorporating non-invasive imaging techniques such as MRI. The development of diffusion-weighted sequences and techniques, such as diffusion tensor imaging (DTI) is currently integrated into MRI medical systems. DTI-based studies also allow the application of a variety of animal models for the study of NDDs (Müller et al.). Further, microscopic tissue examination can also be achieved by DTI at high fields ultra-high fields (Gatto et al., 2018). In a rodent (rat) model of Duchenne muscular dystrophy (DMD), investigators used DTI and high-resolution Localized MR spectroscopy (MRS) to study the brain and temporalis muscle structure *in-vivo*. Imaging findings of this study indicated a disturbed motor and sensory signaling, resulting in dysfunctional neurotransmission, as well unstable osmoregulation in this genetically modified preclinical brain tissue (Xu et al.). On the other hand, DTI and 18F-Fludeoxyglucose (FDG) PET were also used to evaluate the effects on morphology and glucose utilization levels during pulsed Focused Ultrasound (pFUS) and microbubbles (MB) sessions in the rat cortex and hippocampus, which can be used as the benchmarks for the future study of NDDs (Tu et al.). In the line of more complex diffusion model, Diffusion Basis Spectrum Imaging (DBSI) was applied to assess axonal loss after transient dexamethasone treatment in optic neuritis (ON) of mice models experimental autoimmune encephalomyelitis (EAE) related to multiple sclerosis (MS). More important, their finding supported the potential use of DBSI as an *in vivo* imaging outcome measure to assess NDDs related pathologies (Lin et al.). Further on, DTI has been also a valuable tool to study the link between tauopathies (AD) and traumatic brain injury (TBI) n P301L mutant-tau-transgenic-pR5-mice. In combination with immunohistochemistry techniques, the results showed that different parameters from the DTI signal were associated with the co-occurrence of tau-phosphorylation and glial activity following TBI (Soni et al.). Recently, X-ray phase-contrast tomography (XPCT), has contributed to the additional description of high-resolution 3D imaging features in AD and MS animal models' vascular tissues (Palermo et al.).

A more comprehensive approach has been the combination of biological models and multi-modal imaging modalities in the investigation of neurological diseases. Multimodal techniques, including magnetization transfer (MT), DTI, and relaxation along a fictitious field (RAFF) in the rotating frame of rank 4 (RAFF4), were used to detect the changes in the myelin content and microstructure (myelin sheets modifications by gliosis) during the remyelination phase by lysophosphatidylcholine (LPC) induced demyelination in the corpus callosum of rats (Holikova et al.). Moreover, multiparametric approaches have been used to assess different aspects of demyelinating (MS) disease in clinical settings (Mustafi et al., 2019).

Preclinical animal models are a fundamental link between the discovery of basic molecular mechanisms from single-cell organisms and full-scale clinical trials. From the acceleration of pharmacological outcomes to the evaluation of feasibility in revolutionary gene therapies, animal models have been making it possible to improve clinical image acquisition procedures and the setup of more comprehensive neuromonitoring protocols. As an example, Parkinson's Disease (PD), a major neurodegenerative disease, is characterized by massive degeneration of dopaminergic neurons in the substantia nigra pars compacta, alpha-synuclein-containing Lewy bodies, and neuroinflammation. In these cases, magnetic resonance (MR) imaging plays a crucial role in the diagnosis and monitoring of disease progression and treatment. A variety of MR methods are available to characterize neurodegeneration and other disease features such as iron accumulation and metabolic changes in PD animal models (Petiet). Neuroimaging changes were also been characterized in a PD patient with excessive daytime sleepiness (PD-EDS), revealing regional hypertrophy of the striatum in the cohort, concluding that this early bioimaging marker would provide valuable information when investigating PD-EDS (Gong et al.).

In this Research Topic, we found that the contribution of new computational approaches, combined imaging techniques, and animal models keeps expanding the neuroscience field and the discovery of new imaging biomarkers of NDDs. Therefore, we provide the reader with a wide-ranging overview of current innovative imaging techniques that are sensitive to novel biological paradigms and animal models to aid translational research in the diagnosis and monitoring of patient populations suffering from these devastating illnesses. Ultimately, this article collection demonstrates the expanding integration of artificial and biological models to improve translational and therapeutical approaches among these exciting and significant fields.

## Author Contributions

Both authors listed have made a substantial, direct, and intellectual contribution to the work and approved it for publication.

## Conflict of Interest

The authors declare that this article collection was conducted in the absence of any commercial or financial relationships that could be interpreted as a potential conflict of interest.

## Publisher's Note

All claims expressed in this article are solely those of the authors and do not necessarily represent those of their affiliated organizations, or those of the publisher, the editors and the reviewers. Any product that may be evaluated in this article, or claim that may be made by its manufacturer, is not guaranteed or endorsed by the publisher.
